# Plasma Homocysteine and Serum Folate and Vitamin B_12_ Levels in Mild Cognitive Impairment and Alzheimer’s Disease: A Case-Control Study

**DOI:** 10.3390/nu9070725

**Published:** 2017-07-08

**Authors:** Fei Ma, Tianfeng Wu, Jiangang Zhao, Lu Ji, Aili Song, Meilin Zhang, Guowei Huang

**Affiliations:** 1 Department of Epidemiology and Biostatistics, School of Public Health, Tianjin Medical University, Tianjin 300070, China; mafei@tmu.edu.cn; 2 Department of Nutrition and Food Science, School of Public Health, Tianjin Medical University, Tianjin 300070, China; wtfjy.love@163.com (T.W.); jilusalina@126.com (L.J.); zhangmeilin@tmu.edu.cn (M.Z.); 3 Community Health Service Center, Sanhuailu Street, Binhai New District, Tianjin 3000450, China; zhaojiangang107@163.com (J.Z.); SongAili@163.com (A.S.)

**Keywords:** mild cognitive impairment, Alzheimer’s disease, folate, homocysteine, case-control study

## Abstract

Homocysteine (Hcy) is a risk factor for brain atrophy, cognitive impairment, and dementia. Vitamin B_12_ and folate are cofactors necessary for the methylation of Hcy. However, there is some debate regarding the differing levels of plasma Hcy and serum folate and vitamin B_12_ among healthy controls, patients with mild cognitive impairment (MCI), and patients with Alzheimer’s disease (AD). This study aimed to evaluate how the levels of plasma Hcy and its biological determinants, folate and vitamin B_12_, are related to MCI and AD in older Chinese adults. This is a case-control study including 112 subjects with MCI, 89 AD patients and 115 healthy controls. Diagnosis of AD was made according to the NINCDS-ADRDA and MCI with modified Petersen’s criteria. Serum folate and vitamin B_12_ concentrations were analyzed by radioimmunoassay, and plasma Hcy was assessed by a high-performance liquid chromatography-fluorescence method. Multivariate analysis of regression was used to examine the odds ratio (OR) of MCI or AD with Hcy or vitamin levels. Results have shown that serum folate and vitamin B_12_ levels were significantly lower, but the plasma Hcy level was higher, in patients with MCI and AD than in healthy controls. Multivariate regression analyses showed that subjects in the lowest folate tertile had significantly higher adjusted ORs for MCI (OR: 3.07; 95% confidence interval [CI]: 1.12, 8.07) and AD (3.42; 95% CI: 1.15, 8.34) compared to subjects in the highest tertile. The highest Hcy tertile was significantly associated with MCI (adjusted OR: 2.81; 95% CI: 1.15, 4.73) and AD (adjusted OR: 3.64; 95% CI: 1.13, 9.04) compared to the lowest tertile. No association existed between low vitamin B_12_ levels and AD or MCI (*p* > 0.05). Low blood levels of folate and vitamin B_12_ and elevated Hcy levels were associated with MCI and AD in older Chinese adults, and the association was stronger for AD.

## 1. Introduction

The prevalence and economic costs of Alzheimer’s disease (AD) are increasing along with the increasing number of older adults in the population [1]. Therefore, it is important to identify modifiable risk factors for this disease. Mild cognitive impairment (MCI) is an intermediate stage in the continuum from normal aging to dementia. Elderly individuals with MCI are at high risk of developing dementia, including AD. Subjects with a diagnosis of MCI appear to constitute a clinical entity that can be characterized for treatment interventions [2].

B vitamins, including folate, vitamin B_2_, vitamin B_6_, and vitamin B_12_ are involved in one-carbon transfer reactions such as methylation, which is necessary for the production of monoamine neurotransmitters, phospholipids, and nucleotides in the brain [3]. Low levels of these B vitamins have been associated with increased homocysteine (Hcy) [4], which is known to have a direct neurotoxic effect [5]. Moreover, several cross-sectional [6,7,8,9] and longitudinal [10,11,12,13] studies have proposed that elevated Hcy levels may be an independent risk factor for impaired cognitive function or AD, although other studies found no significant association between Hcy and cognitive function [14,15,16,17]. Variations in folate, vitamin B_6_, and vitamin B_12_ may explain the relationship between Hcy and cognitive performance. However, whether high Hcy levels and low B-vitamin concentrations play a causal role in the pathogenesis of cognitive disease or are the consequences of an inadequate dietary intake secondary to the illness remains an open issue. Findings on the risk of MCI or AD in relation to elevated Hcy levels in the Chinese population is limited, as a folic acid fortification policy has not yet been mandated.

In the present study, we used a case-control design to examine the associations of B vitamins and Hcy concentrations with older Chinese healthy controls, patients with MCI, and patients with AD.

## 2. Materials and Methods

### 2.1. Study Design and Participants

**Figure 1:**
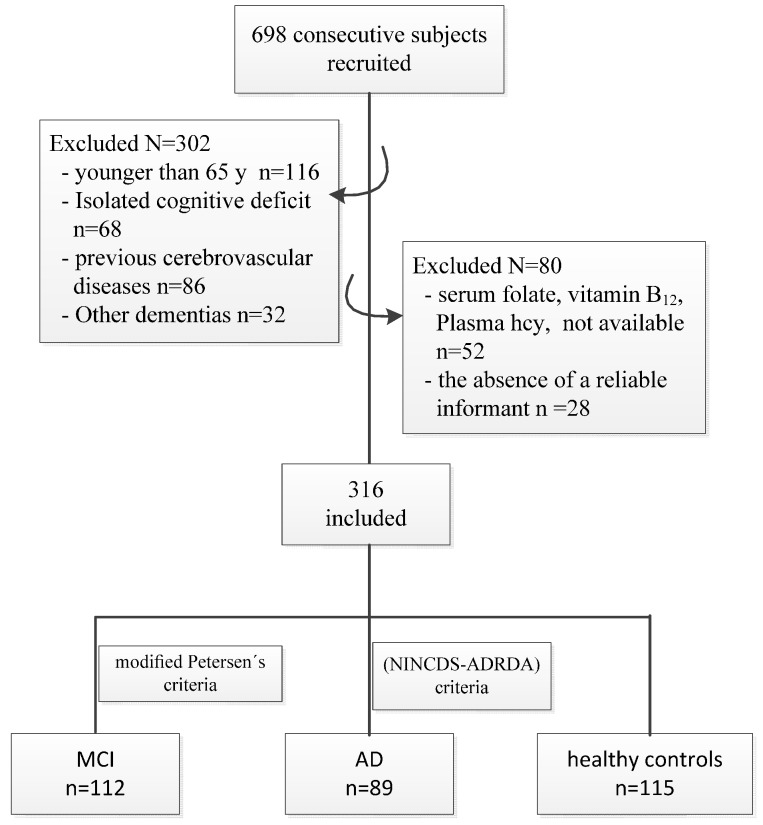
Flow chart detailing the derivation of the study sample.

This was a case-control study designed to evaluate the association of plasma Hcy and its biological determinants, folate and vitamin B_12_, with MCI and AD in elderly Chinese individuals. The recruitment, selection, and classification of patients were performed from April 2014 to June 2014, and a flow chart that outlines this process is shown in Figure 1. A series of 698 consecutive subjects (>65 years of age) were recruited at the neurology departments of several hospitals (Huanhu Hospital, The Second Hospital of Tianjin Medical University and The Sanhuailu Community Health Service Center in Binhai New District) in Tianjin, North China. Of these, 382 individuals were excluded from the analyses because they were younger than 65 years of age (*n* = 116) or because they had an isolated cognitive deficit (*n* = 68) or dementia other than AD (*n* = 32); previous cerebrovascular diseases (transient ischaemic attacks, stroke, or intracranial haemorrhage) (*n* = 86); plasma Hcy, serum folate, and vitamin B_12_ concentrations that were not available (*n* = 52); or the absence of a reliable informant (*n* = 28). The remaining 316 subjects were included in the analysis. All subjects were examined by neurologists and psychiatrists (see “Diagnosis of MCI and AD” for details), and according to their clinical diagnosis, were divided into the following three groups: older healthy control subjects who were free of cognitive impairments (*n* = 115), patients with MCI (*n* = 112), and patients with AD (*n* = 89).

The study was conducted in compliance with the ethical principles of the Declaration of Helsinki. All participants were informed of the objectives of the study and their consent to participate in the study was obtained. The research protocol was approved by the medical ethics committee of Tianjin Medical University, China.

### 2.2. Data Collection

All subjects were interviewed with their caregivers present by trained interviewers. The questionnaire was designed to obtain the following information regarding the patients’ general characteristics: age (in years), sex, education (in years), marital status, smoking status (whether they smoked, the number of packs smoked per year), alcohol use, medical history, medications taken, regular vitamin supplement uptake, and lifestyle habits. As part of the medication use questionnaire, all participants were asked to also report if they were taking any supplements, i.e., vitamins, fish-oils, omega-3 etc., and the frequency, per day. To obtain the amount of folate in each of the reported supplements/vitamins, we collected the ingredients of the supplements (in mg) either from the manufacturer’s website or by contacting the manufacturer. These values were then added to the participants’ nutritional intake data to give the total nutrient intake. The patients’ height and weight were measured, and their body mass index (BMI) (kg/m^2^) was calculated as their weight in kilograms divided by the square of the height in meters (kg/m^2^). Disease duration was defined as the time in years between the onset of the first symptoms (by history) and the clinical diagnosis. Dementia severity was assessed by the clinical dementia rating and the Mini Mental State Examination (MMSE).

### 2.3. Diagnosis of MCI and AD

Dementia was defined based on the clinical criteria of the Diagnostic and Statistical Manual of Mental Disorders, 4th edition (DSM-IV) [18]. The diagnosis of AD was based on the National Institute of Neurological and Communicative Disorders and Stroke and Alzheimer Disease and Related Disorders Association (NINCDS-ADRDA) criteria [19].

Only those individuals who were not diagnosed with dementia were considered for a diagnosis of MCI. The diagnosis of MCI was made by a panel of specialists who reviewed all of the existing information and used the modified Petersen’s criteria [20] as follows: subjective memory complaints with at least a 2-week duration; symptoms were not severe enough to fulfill the DSM-IV criteria for dementia; the cognitive performance was 1.5 standard deviations (SD) below the age-corrected (and education, where available) norms on at least one test in the neuropsychological battery; and activities of daily living (measured by a score < 26) were essentially preserved. The control subjects had no active medical therapy and no personal or family history of neurological and psychiatric disorders, as determined by clinical interviews. They performed within the normative range and did not meet the criteria for MCI or dementia. Based on the results of these evaluations, the participants were classified into the three groups mentioned above.

### 2.4. Blood Sampling and Laboratory Tests

Following overnight fasting (12–14 h), blood samples were collected from each participant. The samples were drawn by venipuncture into 5-mL plain evacuated tubes and then centrifuged at 2000× *g* for 10 min. Serum was used for the analysis of folate and vitamin B_12_ levels, and plasma was used for the analysis of Hcy. All specimens were collected and analyzed within 1 h or stored at −80 °C until use.

The concentrations of folate and vitamin B_12_ were determined on the same day using the Abbott Architect-i2000SR automated chemiluminescence immunoassay system and its supporting kit (Abbott, Washington, NJ，USA). In this assay, folate was quantified by measuring the population of unoccupied folate binding protein sites bound to the matrix using a conjugate of pteroic acid (folate analog) and alkaline phosphatase, as the signal-generating molecule, and a substrate, 4-methylumbelliferyl phosphate. Similar to folate, the serum level of vitamin B_12_ was measured using an Abbott kit based on a microparticle enzyme immunoassay. The concentrations of plasma Hcy were determined by a Hitachi 7180 automatic biochemistry analyzer (Hitachi, Tokyo, Japan), using the enzymatic conversion method. The kit was supplied by Beijing Strong Biotechnologies, Inc. (Beijing, China).

Folate concentrations <3 ng/mL in the serum have traditionally been considered a sign of inadequate folate [21,22]. According to the instruction of Abbott Laboratories, the reference range for serum folate was 7.0–31.4 ng/mL, suggesting that the desirable range for blood folate in the elderly may need to be changed in order to reduce the incidence of neurodegeneration. Other research set the normal and low reference values for folate at >7.2 and <7.2 ng/mL [23], respectively; therefore, we divided the serum folate levels into the following groups: <3 ng/mL, 3–7 ng/mL, and >7 ng/mL. The normal, borderline, and low reference values for vitamin B_12_ were >271, 208–271, and <208 pg/mL, respectively [24]. The normal, moderate, and severe Hcy values were classified as <30, 30–100, and >100 µmol/L, respectively [25].

### 2.5. Statistical Analysis

Data are expressed as the mean and standard deviation. Baseline values were compared among the healthy control, MCI, and AD groups using an analysis of variance (ANOVA) followed by Dunnett’s test or with a chi-squared test followed by a Bonferroni-corrected pairwise comparison test. The vitamin B_12_, folate, and Hcy concentrations were log-transformed before the analyses, as their distributions were slightly skewed. The cumulative frequency distributions of the Hcy and folate concentrations in patients with MCI and AD were compared with those in healthy control subjects by using the Kolmogorov–Smirnov test. Multiple logistic regression analyses were used to estimate the associations between quintiles of plasma Hcy, serum folate, and serum vitamin B_12_ concentrations and MCI and AD. Reference tertiles were the top tertiles for folate and vitamin B_12_ and the bottom tertile for plasma Hcy. The MCI and AD groups were each compared against the healthy control group in all analyses involving odds ratios (ORs). The crude ORs of MCI and AD were estimated using univariate logistic regression analyses in the three tertile levels of vitamin B_12_, folate, and Hcy. Folate, vitamin B_12_, and Hcy, together with age, sex, and education, were entered as covariates into multivariate logistic regression analyses to estimate the adjusted ORs of MCI and AD in the three tertiles of folate, vitamin B_12_, and Hcy. All analyses were performed using SPSS PASW Statistics for Windows, version 18.0 (SPSS Inc., Released 2009, Chicago, IL, USA).

## 3. Results

The demographic, clinical, and biochemical characteristics of the three groups (healthy controls, MCI, and AD) are shown in Table 1. The groups were well balanced in terms of their demographic and clinical information. The mean serum folate and vitamin B_12_ levels were significantly lower in the MCI and AD groups than they were in the healthy control group (*p* < 0.05). The mean plasma Hcy levels were significantly higher in patients with MCI and AD than they were in healthy controls (*p* < 0.01). The AD group had significantly lower mean serum folate and vitamin B_12_ levels and higher mean Hcy concentrations than did the MCI group (*p* < 0.05).

**Figure 2:**
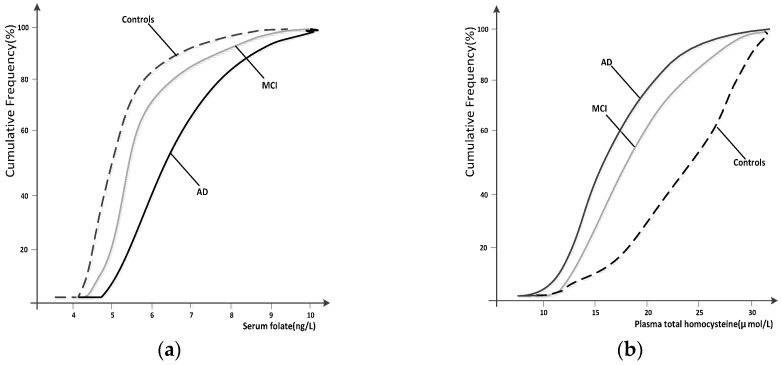
Cumulative frequency distributions of serum folate (**a**) and plasma homocysteine (Hcy) (**b**) levels in the healthy controls, MCI and AD subjects.

The cumulative frequency distributions of the folate and Hcy concentrations in the three groups are shown in Figure 2. The cumulative frequency of both folate and Hcy in the MCI group grew together with that in the AD group, which appeared distinctly separate in the healthy control group. The cumulative frequency plots show a shift in the distribution of the Hcy concentrations to higher values in patients with MCI and AD compared with healthy controls. There was a marked shift in the distribution of the folate concentrations to lower values in both patients with MCI and AD compared with healthy controls.

**Figure 3:**
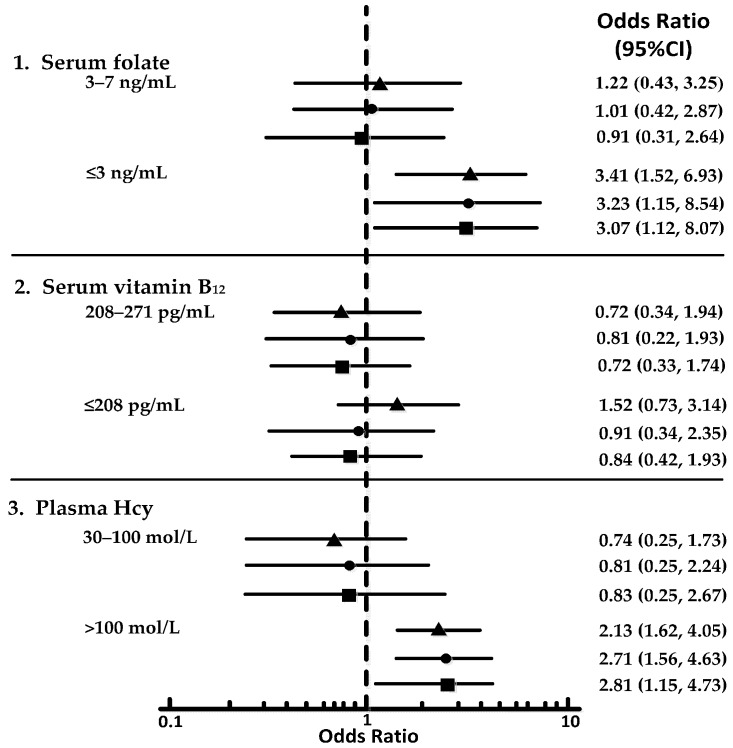
Forest plots showing ORs for tertiles of the vitamin and Hcy levels in MCI. 1 indicates comparing to tertile of >7 ng/mL; 2 indicates comparing to tertile of >271 pg/mL; 3 indicates comparing to tertile of ≤30 mol/L. This figure illustrates unadjusted model (▲), adjusted model for age, sex, and education (●); and adjusted model for age, sex, education, folate, vitamin B_12_, and Hcy (■).

**Figure 4:**
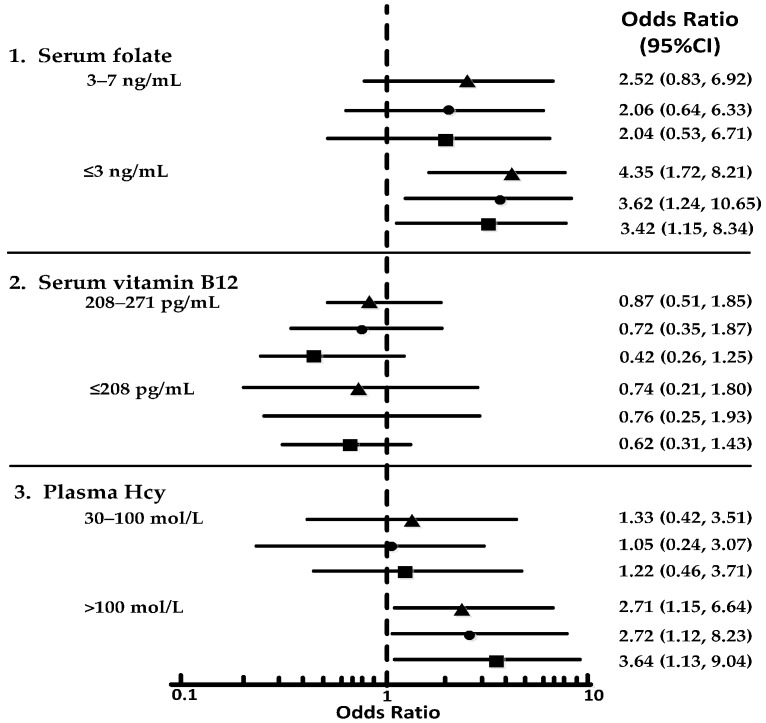
Forest plots showing ORs for tertiles of the vitamin and Hcy levels in AD. 1 indicates comparing to tertile of >7 ng/mL; 2 indicates comparing to tertile of >271 pg/mL; 3 indicates comparing to tertile of ≤30 mol/L. This figure illustrates unadjusted model (▲), adjusted model for age, sex, and education (●); and adjusted model for age, sex, education, folate, vitamin B_12_, and Hcy (■).

The crude (unadjusted) and adjusted ORs for the MCI and AD groups according to the tertile concentrations of serum folate, serum vitamin B_12_, and plasma Hcy are shown in forest plots (Figure 3 and Figure 4). MCI and AD were each significantly associated with the lowest tertile of folate and the highest tertile of Hcy. However, no association emerged between low vitamin B_12_ levels and AD or MCI. The lowest folate tertile, compared to the highest tertile, was associated with both MCI (crude OR: 3.41; 95% confidence interval [CI]: 1.52, 6.93) and AD (crude OR: 4.35; 95% CI: 1.72, 8.21). Compared to the lowest tertile, the highest Hcy tertile was associated with MCI (crude OR: 2.13; 95% CI: 1.62, 4.05) and AD (crude OR: 2.71; 95% CI: 1.15, 6.64). These ORs did not vary significantly when adjusted for age, sex, and education or when folate and vitamin B_12_ or Hcy concentrations were included as covariates in the model: subjects in the lowest folate tertile had an adjusted OR of 3.07 (95% CI: 1.12, 8.07) for MCI and of 3.42 (95% CI: 1.15, 8.34) for AD. Subjects in the highest Hcy tertile had an adjusted OR of 2.81 (95% CI: 1.15, 4.73) for MCI and of 3.64 (95% CI: 1.13, 9.04) for AD. In the MCI group, the chi-squared tests that were conducted before the individual OR comparisons showed nearly significant differences among the three folate tertiles in the crude analysis (*p* = 0.042), adjusted model 1 (*p* = 0.031), and adjusted model 2 (*p* = 0.037). In the AD group, all of the comparisons were significant among the folate and Hcy tertiles based on the chi-squared test.

To assess whether the prior duration of AD could explain the observed biochemical changes, 89 patients with AD with available data were classified by tertiles of duration of memory impairment (as reported by an informant) before the blood samples were taken (Table 2). The disease severity (MMSE score) was substantially greater in those with a longer duration of memory impairment, but there was no significant trend in the mean levels of any of the biochemical variables with the increasing duration of symptoms. The biochemical findings were also unaltered by the illness duration among patients with MCI (data not shown).

## 4. Discussion

In the present study, we retrospectively identified that MCI and AD were significantly associated with high plasma Hcy concentrations and low serum folate concentrations. In the highest Hcy tertile and the lowest folate tertile, the risk of MCI or AD was two to three times greater than the risk in subjects in the lowest and highest tertiles, respectively. A stronger association was shown in patients with AD compared to in patients with MCI.

Hcy may exert its neurotoxic effects by activating the *N*-methyl-d-aspartate receptor, leading to cell death [26], or by being converted into homocysteic acid, which also has an excitotoxic effect on neurons [27]. An Hcy-lowering effect in patients with AD has also been shown in an open-label trial using a folic acid, vitamin B_12_, and vitamin B_6_ regimen [28]. The association of low folate and vitamin B_12_ levels with AD may be mediated by the effects that folate and vitamin B_12_ have on Hcy levels [29], or may be related to their effects on methylation reactions in the brain [30]. Future randomized clinical trials with Hcy-reducing therapies should be conducted to provide further evidence of the relationships among Hcy, B vitamins, and cognition.

In agreement with the findings of the Kungsholmen population-based study [31] and the Bronx Longitudinal Aging Study [32], we did not find any significant association between the serum vitamin B_12_ concentrations and MCI and AD. The associations among vitamin B_12_, cognitive dysfunction, and AD in older individuals are weaker than are the folate associations [6,33,34,35], and often, the folate associations are absent or nonsignificant [8,31,32,33,34,35,36,37]. A better measure of vitamin B_12_ status may be the level of holotranscobalamin, which is an early marker of vitamin B_12_ deficiency. We hope to test this in a future prospective study.

Subjects with MCI have a high risk of developing AD in the short term or are already in a preclinical phase of dementia [38]. Thus, by comparing the biochemical profile of subjects with MCI with the profiles of the healthy control and AD groups, we tried to shed light on the possible role of folate, vitamin B_12_, and Hcy in the prodromal phases of the disease. The results of the current study suggest that the MCI group had both a lower mean folate concentration and a higher mean Hcy concentration compared to the healthy control group. These associations were independent of known or putative risk factors and were not modified by further adjustments for vitamin B_12_ and folate or vitamin B_12_ and Hcy.

The cumulative frequency plots showed that the frequency distribution of vitamin B_12_ in the MCI group was very similar to the distribution observed in the AD group. Interestingly, as was found in the study by Clarke et al. [7], the cumulative frequency distributions of the folate concentrations were more markedly separated than were the cumulative frequency distributions of the Hcy concentrations. However, we did identify a significant decrease in the serum folate levels in AD < MCI < healthy controls and a significant increase in the Hcy concentrations in AD > MCI > healthy controls (Figure 2). Additional studies are needed to establish the significance of these associations.

Some limitations of our study need to be addressed. First, this was a case-control study, and thus it cannot prove causality. It could, for example, be argued that AD leads to reduced serum folate and vitamin B_12_ concentrations, causing an elevation in Hcy levels. We cannot refute this possibility in this case-control study. Therefore, our findings need to be confirmed in further longitudinal studies. Second, as a random sample was not utilized, it is unlikely that our study population is representative of the general elderly population. Thus, our results may be difficult to interpret and apply to the general population. A further limitation of this study is the lack of data on the recent dietary intake and vitamin supplements in patients compared to in healthy controls.

Despite the above limitations, the chief strengths of the present study are as follows: (1) this was a relatively large, rigorous, population-based study designed to evaluate the association of plasma Hcy and its biological determinants, folate and vitamin B_12_, with MCI and AD in China; (2) the comprehensive clinical evaluations and the consensus approach provided reliable diagnoses of MCI and AD; and (3) the availability of pre-study plasma Hcy levels and baseline values for serum B vitamins and other covariates.

## 5. Conclusions

Based on the findings of the present study, it can be concluded that the low levels of folate and high levels of Hcy may be associated with a higher risk of MCI and AD. Because associations are not proof of a causal relationship, large, randomized, controlled clinical trials on B vitamin supplementation (a combination of folate, vitamin B_12_, and vitamin B_6_) and the onset and course of MCI and AD are under way.

## Figures and Tables

**Table 1:** Demographic, clinical, and biochemical characteristics of the three groups.

Profile	Control Group (*n* = 115)	MCI Group (*n* = 112)	AD Group (*n* = 89)
Demographics			
Age (years)	72.82 ± 8.87	73.23 ± 8.67	74.62 ± 8.01
Female	69 (60.00)	68 (60.71)	56 (62.92)
Total education (years)	10.92 ± 1.53	10.42 ± 2.01	10.33 ± 2.37
Clinical characteristics			
Disease duration (months)			27.67 ± 22.24
MMSE score	28.53 ± 1.74	16.23 ± 8.07 ^‡^	12.83 ± 8.13 ^‡^
BMI (kg/m^2^)	25.87 ± 5.13	25.14 ± 3.96	24.73 ± 5.54
Current smokers	24 (20.87)	24 (21.43)	20 (22.47)
Alcohol (units/week)	9.12 ± 4.53	8.14 ± 3.22	8.57 ± 3.21
Total cholesterol (mmol/L)	5.83 ± 1.35	6.02 ± 1.36	5.74 ± 1.43
Systolic blood pressure (mmHg)	143.33 ± 25.20	143.31 ± 25.21	144.44 ± 26.47
Hypertension	50 (43.48)	49 (43.75)	38 (42.70)
Diabetes	20 (17.39)	21 (18.75)	16 (17.98)
Biochemical measures			
Plasma Hcy (μmol/L)	13.21 ± 4.05	15.35 ± 8.44 ^‡^	16.37 ± 7.46 ^‡^
Serum folate (ng/mL)	7.03 ± 3.68	5.74 ± 2.63 ^†^	5.13 ± 3.57 ^†^
Serum vitamin B_12_ (pg/mL)	573.17 ± 75.41	538.82 ± 84.06 ^†^	531.21 ± 44.33 ^†^
B vitamin supplements, *n* (%) *	31 (26.96)	31 (27.68)	23 (25.84)
Use of fish-oils, omega-3, *n* (%)	16 (13.91)	16 (14.28)	12 (13.48)

Results are shown as *n* (%) for the chi-squared or Fisher’s exact tests, and as the mean ± the standard deviation for independent *t*-tests (two-tailed) or analyses of variance. The vitamin B_12_, folate, and Hcy concentration values were log-transformed before the analyses. MCI, mild cognitive impairment; AD, Alzheimer’s disease; BMI, body mass index; MMSE, Mini-Mental State Examination; Hcy, homocysteine. ^†^ *p* < 0.05 vs. healthy controls; ^‡^ *p* < 0.01 vs. healthy controls. * Intake of multivitamin supplements or any type of B vitamin supplements.

**Table 2:** Clinical and biochemical variables in patients with AD by duration of memory impairment at presentation.

Tertiles of Duration of Memory Impairment, Years	MMSE Score	Biochemical Variables		
		Hcy, μmol/L	Folate, ng/mL	Vitamin B_12_, pg/mL
I <2	17.80 ± 7.99	17.25 ± 6.82	5.67 ± 4.11	525.31 ± 37.21
II 2–4	14.22 ± 8.44	16.47 ± 5.75	5.11 ± 5.17	528.47 ± 63.41
III >4	7.55 ± 6.49	16.79 ± 4.15	5.05 ± 7.39	517.45 ± 43.49
*p* value	0.007	0.452	0.321	0.367

Results are shown as the mean ± the standard deviation for analyses of variance. AD, Alzheimer’s disease; MMSE, Mini-Mental State Examination; Hcy, homocysteine.

## Abbreviations

The following abbreviations are used in this manuscript:

AD	Alzheimer Disease
ANOVA	Analysis of Variance
BMI	Body Mass Index
DSM-IV	Diagnostic and Statistical Manual of Mental Disorders, 4th edition
Hcy	Homocysteine
IQ	Intelligence Quotient
MCI	Mild Cognitive Impairment
MMSE	Mini Mental State Examination
NINCDS-ADRDA	National Institute of Neurological and Communicative Disorders and Stroke and Alzheimer Disease and Related Disorders Association
ORs	Odds Ratios
SD	Standard Deviations
WAIS-RC	Chinese version of the Wechsler Adult Intelligence Scale-Revised
